# Acute Traumatic Intracerebral Hematoma: To Operate or Not to Operate?

**DOI:** 10.7759/cureus.98442

**Published:** 2025-12-04

**Authors:** Lukasz Grabarczyk, Aleksandra Grosz, Grazyna Waska, Kamil Sobolewski, Pawel Radkowski

**Affiliations:** 1 Department of Neurological Surgery, Alarm Clock Clinic, Coma Recovery and Neurorehabilitation Center, Warsaw, POL; 2 Faculty of Medicine, Medical University of Łódź, Łódź, POL; 3 Department of Internal Medicine, Specialist Hospital No. 1 in Bytom, Bytom, POL; 4 Department of Anesthesiology and Intensive Care, Municipal Polyclinical Hospital, Olsztyn, POL; 5 Department of Anesthesiology and Intensive Care, Collegium Medicum University of Warmia and Mazury in Olsztyn, Olsztyn, POL

**Keywords:** conservative medical management, intracranial hemorrhage, medical intensive care unit (micu), severe combined traumatic brain injury, traumatic intracerebral hematoma

## Abstract

Acute traumatic intracerebral hematoma (TICH) is a severe complication of head injury, often associated with high morbidity and mortality. We report the case of a 44-year-old man admitted to the ED after a craniocerebral trauma caused by a fall from a tractor. On admission, he presented with impaired consciousness, scoring 8 on the Glasgow Coma Scale, with eye opening to pain (E2), incomprehensible verbal responses (V2), and withdrawal to painful stimuli (M4). Seizures and hemodynamic instability were also noted. CT revealed a large left frontal lobe hematoma with intraventricular and subarachnoid hemorrhage. In cooperation with the neurosurgical team, a decision was made to initiate conservative treatment in the ICU, focusing on airway protection, intracranial pressure control, hemodynamic stabilization, seizure prophylaxis, and infection prevention. During the following days, gradual clinical improvement was observed, with restoration of consciousness and regression of neurological deficits. Serial imaging demonstrated progressive resorption of the hematoma. The patient was discharged after 17 days in good general condition and transferred for rehabilitation, with only mild residual right lower limb weakness and minor cognitive impairment. This case highlights that, in selected patients, conservative management of TICH may lead to a favorable outcome despite extensive hemorrhage.

## Introduction

Acute traumatic intracerebral hematoma (TICH) is most often caused by a traumatic brain injury. The term describes bleeding into the brain tissue as a result of disruption of the blood vessel endothelium [1,2]. In the initial phase of injury, rupture of a blood vessel leads to the formation of a hematoma, which has a mass effect, compressing the brain parenchyma and disrupting its structure. This leads to the release of substances that damage the blood-brain barrier, causing brain swelling and apoptosis of neurons and glial cells [3]. Survival and recovery after intracerebral hemorrhage (ICH) depend on the location and size of the hematoma, its mass effect, and the increase in intracranial pressure. In addition, prognosis is influenced by secondary injury mechanisms, such as cerebral edema, inflammatory responses, and complications resulting from prolonged neurological dysfunction [4]. The clinical picture of these hematomas is highly variable and can include sudden disturbances of consciousness, focal neurological deficits such as hemiparesis, aphasia, and ataxia, as well as symptoms of intracranial hypertension, including headache, vomiting, and papilledema. Unfortunately, this condition is associated with high patient mortality and morbidity despite advanced treatment. However, we report here a successful case of conservative treatment in a 44-year-old man with TICH.

## Case presentation

**Figure 1:**
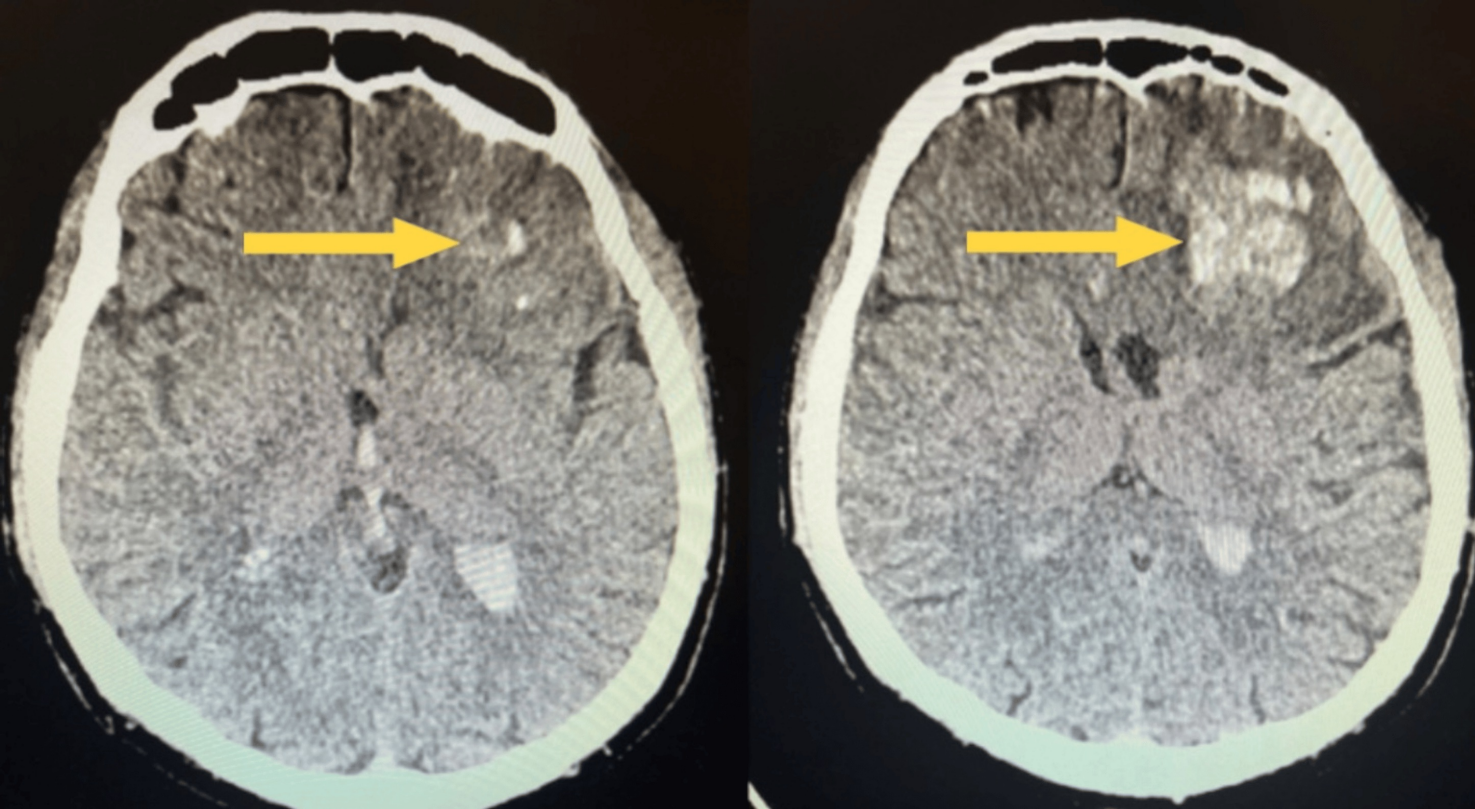
CT scan of the brain performed on the first day of hospitalization Non-contrast CT brain scan showing a large left frontal intracerebral hematoma with intraventricular and subarachnoid extension (arrow).

A 44-year-old man was admitted to the ED with a craniocerebral injury sustained from a fall from a tractor, followed by a brief loss of consciousness and two epileptic seizures. On admission, he was profoundly confused, with a limited response to stimuli (Glasgow Coma Scale score: 8 points - eye opening 2, verbal response 2, motor response 4), and showed cardiovascular and respiratory instability. A CT scan revealed an extensive left frontal lobe intracerebral hematoma measuring 62 × 45 × 58 mm, with evidence of extension into the ventricular system: blood was present in the body and occipital horn of the left lateral ventricle, in the occipital horn of the right lateral ventricle, in the third ventricle, within the cerebral vasculature, and subarachnoidally in the fourth ventricle, in the sulci of both cerebellar hemispheres, and periaqueductally (Figure 1). The midline shift at this initial CT was measured at 11 mm. Despite the large hematoma and intraventricular extension, surgical intervention was not indicated due to a stable neurological examination, absence of herniation, and controlled intracranial pressure, supporting a nonoperative management strategy.

Due to the absence of significant intracranial hypertension and a mass effect primarily in the left frontal lobe, without notable displacement of brain structures, a decision was made, in consultation with the neurosurgical team, to pursue conservative treatment in the ICU. The patient had been intubated in the ED following severe head trauma, and mechanical ventilation was continued after transfer to the ICU. The main therapeutic goals included prevention of arterial hypotension, maintenance of normal intracranial pressure, and prevention of hypoxia, hypoglycemia, electrolyte disturbances, coagulation disorders, infections, and epileptic seizures.

The patient received conservative treatment for 17 days post-injury. Analgosedation was maintained with midazolam (20-60 mg/24 h), morphine, and propofol (1.5-3 mg/kg/h), along with anti-edema therapy using 20% mannitol (1.2 g/kg body weight) and 5% NaCl (2.5 ml/kg body weight). Empiric antibiotic therapy with ceftriaxone and metronidazole was administered in response to a positive endotracheal tube culture, which grew *Pseudomonas aeruginosa *and *Candida albicans*. Therapy was guided by microbiological results in accordance with standard ICU infection protocols. Diuresis was stimulated using torasemide at doses of 0.2-0.4 mg/kg body weight. While specific numerical targets for parameters such as blood pressure, PaCO₂, sodium, and fluid balance were not formally documented, all management decisions were guided by the “6×N” principle of neurocritical care, maintaining normothermia, normotension, normoglycemia, normoventilation, normocardia, and normovolemia, ensuring stable physiological conditions throughout the ICU stay.

**Figure 2:**
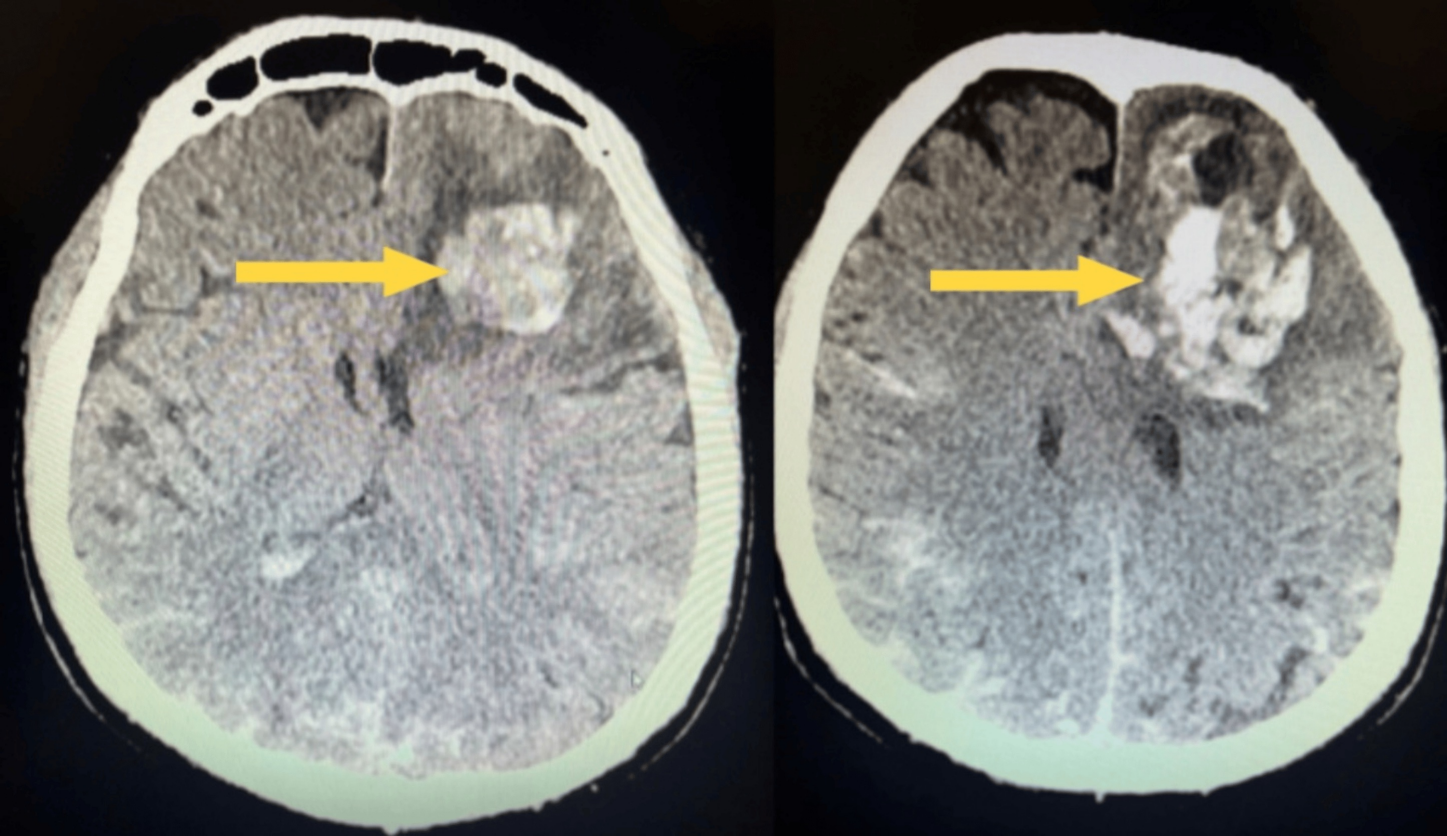
Follow-up CT scan of the head performed on the second day after injury Follow-up CT scan on day 2 showing increased white matter edema with greater mass effect and subarachnoid hemorrhage with abundant blood in the parietal and occipital sulci.

A follow-up CT scan performed on the second day revealed slightly increased hypotensive white matter edema with greater mass effect, narrowing of the fissures, and compression of the frontal horns of the lateral ventricles, but without clear signs of subfalcine herniation. Additionally, subarachnoid hemorrhage was observed, with large amounts of blood in the sulci of the parietal and occipital lobes (Figure 2). The patient remained unconscious in a critically severe general condition.

The patient remained under close observation and continued conservative treatment. On day 7, he regained consciousness, followed simple commands, and maintained logical contact. Doses of morphine and midazolam were gradually reduced. He continued to experience a fever ranging from 37.5 °C to 39.5 °C.

A follow-up CT scan of the head was performed on day 9. Compared with the initial scan, which showed an intracerebral hematoma measuring 65 × 42 × 58 mm, the repeat imaging demonstrated significant regression of the hematoma to approximately 32 × 18 mm, accompanied by partial resorption of the clotted blood and a reduction in the displacement of the brain ventricles.

**Figure 3:**
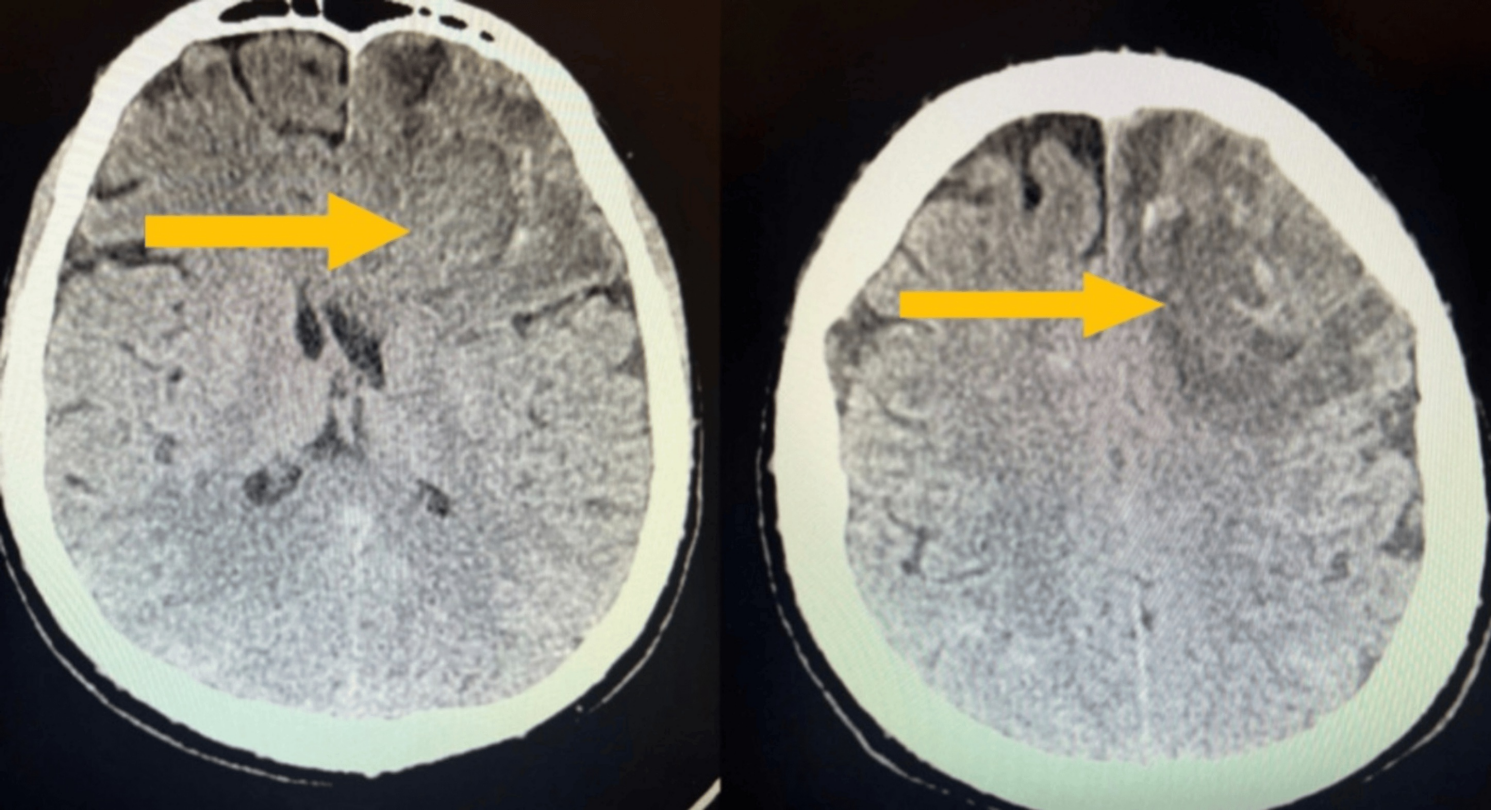
Follow-up CT scan of the head on day 15 of treatment Follow-up CT on day 15 showing marked hemolysis of the left frontal hematoma with surrounding edema and residual mild subarachnoid hemorrhage in the posterior parietal and occipital sulci.

Before extubation, sedation and ventilatory support were gradually reduced as part of the weaning process. On day 12, the patient was successfully extubated and remained in logical verbal contact, oriented to place and time. Dexmedetomidine was used for sedation, with the dose gradually reduced over the following days of the patient's stay in the ICU. On neurological examination, there was no muscular paresis or neck stiffness; muscle strength was symmetrical, and pupils were symmetrically responsive to light. On day 15, a follow-up CT scan of the head showed significant hemolysis of the left frontal lobe intracerebral hematoma, with a hemorrhagic area measuring 25 × 15 mm, surrounded by a zone of edema and forming a small mass effect. Subarachnoid hemorrhage was much less severe, mainly located in the sulci of the posterior parietal and occipital lobes (Figure 3).

**Figure 4:**
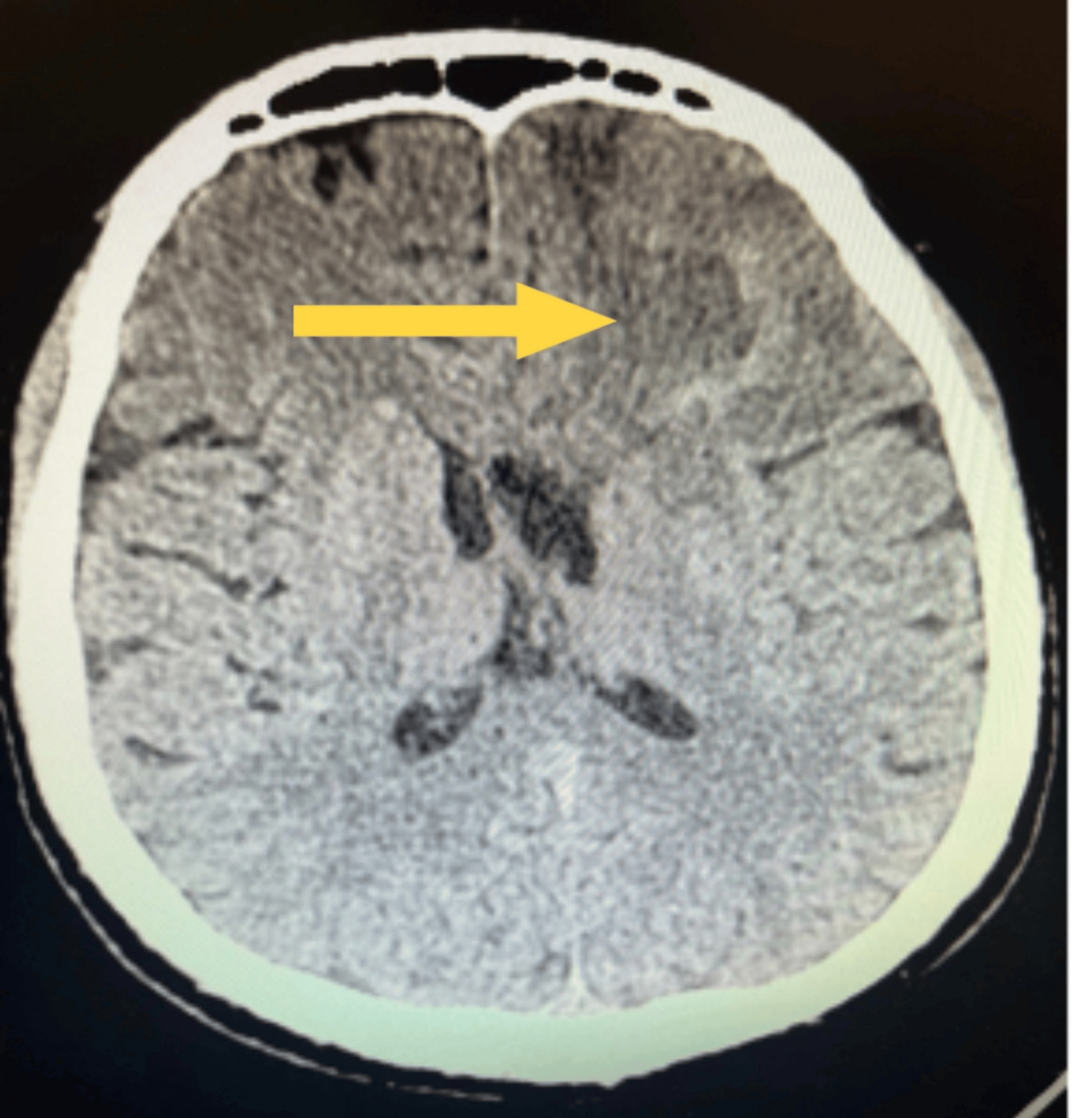
Follow-up CT scan of the head on day 22 of treatment Follow-up CT on day 22 demonstrating complete resorption of the intracerebral hematoma with cortical-subcortical atrophy and mild ventricular dilatation.

On post-injury day 17, the patient was discharged in good general condition, with stable respiratory and circulatory function, for further rehabilitation in the neurology department. After a week of intensive motor rehabilitation in the unit, the patient was discharged home. On the day of discharge, neurological examination revealed no cranial nerve deficits, no nystagmus, normal coordination tests, and no deep or superficial sensory disturbances. The only finding was muscle weakness of the right lower limb, graded 4/5 on the Lovett scale; the other limbs were normal. Neuropsychological examination, following organic changes in the central nervous system, showed slight impairments in cognitive function, memory, and behavioral variability. A follow-up CT scan of the head performed on post-injury day 22 showed complete resorption of the intracerebral hematoma and features of cortical-subcortical atrophy with mild dilatation of the ventricular system (Figure 4). Neurological examination at this time revealed full recovery of muscle strength in the right lower limb (5/5 on the Lovett scale), reflecting progressive clinical improvement during hospitalization and consistent with the radiological resolution of the hematoma. Overall, the patient maintained stable cognitive and motor function, was able to move independently, and was discharged home under family supervision.

## Discussion

**Figure 5:**
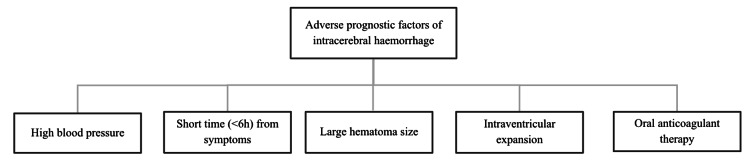
Adverse prognostic factors of ICH ICH, intracerebral hemorrhage

The main objective in the treatment of ICH is to stop active bleeding as soon as possible, as this is a key prognostic factor affecting patient outcomes [5]. Other important prognostic parameters include elevated blood pressure, early therapeutic window (i.e., less than six hours from symptom onset), initial hematoma volume (moderate to large), blood penetration into the ventricular system, and the use of oral anticoagulant therapy (Figure 5) [6,7]. In this case, the intracerebral hematoma measured 62 × 45 × 58 mm on admission and showed progressive resorption, with complete resolution by day 22. The reduction in hematoma size closely correlated with clinical recovery, including restoration of full muscle strength and cognitive function.

ICH arises from multiple etiological mechanisms, and common categories along with their specific causes are summarized for reference (Table 1). Primary therapeutic considerations focus on the prevention of secondary brain tissue damage, implementation of comprehensive rehabilitation, and the prevention of recurrent ICH and cardiovascular ischemic complications. In selected cases, surgical intervention may be indicated, particularly for patients with large or life-threatening hematomas, significant mass effect, increased intracranial pressure, or rapid neurological deterioration. The decision for surgery should be individualized, taking into account hematoma size, location, patient comorbidities, and overall clinical status.

**Table 1:** Etiological categories and common causes of ICH ICH, intracerebral hemorrhage

Etiological category	Specific reasons
Hypertension	Chronic hypertension causing microangiopathy and vascular rupture
Vascular pathologies	Cerebral aneurysms, arteriovenous malformations, and amyloid angiopathy
Hemostatic disorders	Hemorrhagic diathesis (congenital and acquired), thrombocytopenia, and hemophilia
Antithrombotic and antiplatelet pharmacotherapy	Oral anticoagulants (warfarin and non-vitamin K antagonist oral anticoagulants) and anti-aggregation drugs (acetylsalicylic acid and clopidogrel)
Craniocerebral injuries	Mechanical injuries resulting in intracranial hematomas
Brain tumors	Primary and metastatic tumors with a hemorrhagic component
Infection and inflammation	Cerebral vasculitis, nervous system infections, and septicemia
Exogenous and idiopathic factors	Alcohol abuse, psychoactive substances (cocaine and amphetamines), and hemorrhages of undetermined origin

The Early Minimally Invasive Removal of Intracerebral Hemorrhage (ENRICH) study compared the efficacy of minimally invasive transcranial parafascicular surgery combined with pharmacological treatment according to guidelines with conservative treatment alone. The results showed that therapy combining surgical intervention and conservative management produced better outcomes than pharmacological management alone [8].

The INTERACT3 study demonstrated that the use of a care protocol combining intensive blood pressure lowering with other physiological control strategies, implemented within hours of symptom onset, improved functional outcomes in a broad range of patients with acute ICH [9].

The randomized ANNEXA-I trial evaluated the efficacy of andexanet alfa compared with standard care in patients with acute intracranial hemorrhage who were receiving oral factor Xa inhibitors. The results showed that intravenous administration of andexanet alfa, a recombinant modified factor Xa, was superior to standard treatment (including prothrombin complex concentrate in 86% of cases) in terms of hemostatic efficacy [10].

The latest results from the fourth INTEnsive ambulance-delivered BP Reduction in hyper-ACute stroke Trial (INTERACT4 trial) emphasize the importance of rapid blood pressure control within hours of intracranial hemorrhage onset [11].

Conceptually, early removal of a blood clot from the brain could provide significant benefits to patients with intracranial hemorrhage, similar to the effects of mechanical thrombectomy in ischemic stroke [12]. Nevertheless, pivotal studies evaluating different interventional methods have shown neutral results, although a systematic review and meta-analysis suggested potential benefits.

The choice between conservative and surgical management in ICH largely depends on several factors, including clot size, location, patient clinical status, and timing of intervention. Large or life-threatening hematomas, significant mass effect, rapidly deteriorating neurological function, or increased intracranial pressure may warrant surgical evacuation, whereas small to moderate hematomas in stable patients are often managed conservatively with close monitoring, supportive care, and early rehabilitation. This individualized approach allows optimization of outcomes while minimizing procedural risks.

In the case described here, the patient was treated exclusively conservatively, first in the ICU and then in the stroke and rehabilitation unit. Basic life-sustaining measures for a patient with ICH included ensuring airway patency, prevention of regurgitation, adequate ventilation, and careful control of arterial pressure. The aim was to maintain a mean arterial pressure of approximately 90 mm Hg or a systolic pressure not exceeding 140 mm Hg. Corticosteroids were not used, as they are contraindicated in the treatment of cerebral edema associated with ICH.

Thus, studies to date have not provided conclusive evidence of the superior efficacy of surgical treatment compared with conservative management.

## Conclusions

Conservative management of TICH may be effective in strictly selected patients who do not demonstrate clinical or radiological features of herniation, progressive neurological deterioration, or significant midline shift. In such cases, continuous neuro-critical care monitoring, rigorous control of physiological parameters, tailored intracranial pressure-oriented therapy, and early rehabilitation play a key role in achieving favorable neurological outcomes. Although surgical evacuation remains the standard of care for many patients with large hematomas or intraventricular extension, this case illustrates that a nonoperative approach can be a safe and viable option when clear criteria are met and meticulous surveillance is maintained. This report should therefore be interpreted as an example of individualized decision-making rather than a general recommendation for conservative treatment in TICH.
